# Canine nematode and *Giardia* spp. infections in dogs in Edmonton, Alberta, the “CANIDA” study

**DOI:** 10.1186/s13071-022-05386-5

**Published:** 2022-08-22

**Authors:** Darcy Visscher, Emilie Porter, Sarah Sweet, Donald Szlosek, Stephanie Horr

**Affiliations:** 1 Department of Biology, The King’s University, Edmonton, AB T6B 2H3 Canada; 2 Department of Biological Science, University of Alberta, Edmonton, AB T6G 2E9 Canada; 3 Naturalis Biodiversity Center, Leiden, The Netherlands; 4 IDEXX Laboratories Inc, Westbrook, ME 04092 USA

**Keywords:** Dog, Canine, Parasites, Fecal, Flotation, Coproantigen, Hookworm, Whipworm, Ascarid, Giardia

## Abstract

**Background:**

Canine intestinal parasite prevalence may be influenced by geographical region, age, and health status of the dog. Behaviors such as predation, scavenging, or roaming as well as routine administration of anthelmintics also play a role. The purpose of this study was to evaluate fecal test results using zinc sulfate flotation by centrifugation combined with coproantigen testing directed at protein antigens excreted or secreted by hookworms (*Ancylostoma* spp. *Uncinaria stenocephala*), ascarids (*Toxocara canis, Toxascaris* spp. *Baylisascaris* spp.), whipworms (*Trichuris vulpis*), and *Giardia* spp. during active infection in owned dogs visiting dog parks in Western Canada.

**Methods:**

A total of 774 participants were recruited from Edmonton, Alberta, Canada. Canine fecal samples were collected from seven dedicated off-leash dog parks. Participating dog owners responded to a questionnaire regarding their dogs’ signalment, previous veterinary history, and use of parasite-preventive products. Fecal samples were tested using zinc sulfate centrifugation combined with coproantigen testing.

**Results:**

The overall prevalence of canine intestinal parasites in client-owned dogs was similar to previous studies conducted in the US. Mean age of dogs tested was 4 years, with puppies and older dogs having higher rates of infection than the mean. Fecal flotation centrifugation found 3.2% hookworm, ascarid, whipworm, and *Giardia* spp.-positive infections. Coproantigen testing identified 5.8% positive infections, including all of the above that were detected using fecal flotation centrifugation.

**Conclusions:**

Coproantigen testing detected more hookworm, ascarid, whipworm, and *Giardia* spp.-positive samples in addition to detecting all positive results found using fecal flotation centrifugation. Fecal flotation centrifugation combined with coproantigen testing improves sensitivity over flotation alone and may detect pre-patent or sub-clinical infections in dogs visiting public dog parks.

**Graphical Abstract:**

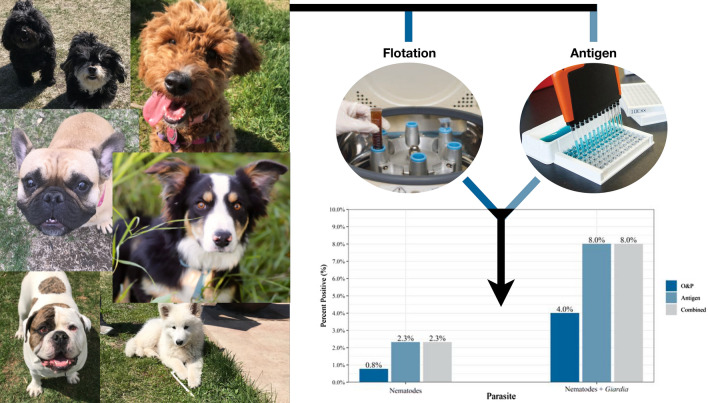

**Supplementary Information:**

The online version contains supplementary material available at 10.1186/s13071-022-05386-5.

## Background

Intestinal parasite testing is recommended 1–4 times a year for adult dogs depending on lifestyle and risk factors and is essential at regular intervals 2–4 times during a puppy’s first year of life [1–3]. The Companion Animal Parasite Council (CAPC) states that combining fecal flotation by centrifugation with coproantigen testing may aid in the identification of intestinal parasites where few to no eggs are recovered, for instance during the prepatent period or in the case of single sex infections. However, widespread adoption of regular intestinal parasite screening protocols remains a challenge, despite studies demonstrating that intestinal parasite infections are a common finding in dogs in the USA [2, 3].

Clinical signs of intestinal parasitism may vary from untreated puppies that present with soft stool and a distended abdomen to adults that may display no overt clinical signs. Some parasites including hookworms and ascarids are zoonotic to humans. While intestinal parasite prevalence in the US has been extensively documented, there are currently fewer studies investigating prevalence of intestinal parasites in Canada.

The purpose of this study was to evaluate fecal test results using zinc sulfate flotation by centrifugation combined with fecal antigen testing for hookworms, ascarids, whipworms, and *Giardia* spp. from dogs visiting dog parks in Western Canada. Owners participated in a brief survey in which they provided information on current and past veterinary care as well as their pets’ diet and exercise habits.

## Methods

### Study area

Participants were recruited from Edmonton, Alberta, Canada. The metropolitan Edmonton area has approximately 1.4 million people and just over 56,000 registered dogs [18, 19]. It has one of the largest urban recreational park systems in North America centered around the North Saskatchewan River which bisects the city (City of Edmonton website). These natural areas are frequently used for recreation, including dog walking, and are home to a resident coyote population. Seven urban dedicated off-leash dog parks (hereafter “dog parks”) were chosen for sampling. Surveys from these parks were collected over at least 3 consecutive days in both early summer (May or June) and then once again in late summer (July and August) of 2020, except for one park which was not revisited in late summer. Ten percent of participants were recruited through an online survey or approached at smaller residential parks that were visited only once. Participants recruited online completed the questionnaire before being met individually for sample collection.

### Questionnaire design

A modified form of the survey tool employed by Smith et al. was used and further divided into different sections [4, 5]. The first section involved participant screening questions for inclusion in the survey. The second section focused on dog demographic details such as gender, breed, age class, spay/neuter status, and veterinary care and deworming practices. The next sections were specific to dog owner behavior including dog walking routines and levels of off-leash activity. Questions regarding dog behavior during walks, including topics such as prey drive and scavenging activity, were also included. A Likert-type ranked 6-point scale (ranging from never to always/daily) was used to record the frequency of walking behavior at different types of locations as well as the frequency of off-leash activity at those locations. To be included in the study, participants were required to agree to answer screening questions, complete the entire survey, and provide a fecal sample. The project received ethics approval by The King’s University ethics board (2017–08-DRV).

### Fecal analysis

Participants were asked to collect a fresh fecal sample from their dog and return it to the researcher before leaving the park. Fecal samples were stored in coolers until the end of the day before being processed into 1.5 ml centrifuge tubes and frozen at − 18 °C on the same day as collected until they were sent for analysis. While awaiting transfer, samples were held at − 80 °C for at least 72 h to inactivate any virulent *Echinococcus* eggs that could be present in the samples [6]. Samples were transferred to the IDEXX Reference Laboratory in Sacramento, CA, USA. The samples were tested using fecal flotation with centrifugation with zinc sulfate (specific gravity 1.24–1.27) as well as hookworm, ascarid, whipworm, and *Giardia* spp. coproantigen immunoassays. The coproantigen immunoassays in this study used polyclonal and monoclonal antibodies developed by IDEXX Laboratories against recombinantly expressed proteins for *T.canis, A.caninum, T.vulpis*, and *Giardia* spp. Sensitivity, specificity, and limits of detection have been previously described [7, 15, 16]. Samples were also tested using real-time polymerase chain reaction targeting both *Echinococcus* spp. and *E. multilocularis* (IDEXX Laboratories *Echinococcus* RealPCR™ Panel) using proprietary forward and reverse primers and hydrolysis probes.^17^

### Statistical Analysis

Fecal samples were tabulated by count and proportion positives for flotation, coproantigen, or both methods combined (either/or could be positive). Questionnaire data were tabulated as the frequency and proportion of responses. Tests for paired proportions were done using the mid-p McNemar exact test. Due to the small percentage of positive parasites, a Firth Penalized logistic regression was used to estimate the odds of having (any) positive parasite found with the following questionnaire covariates: Does your dog chase wildlife on walks or at home? (Yes, reference: No). Has your dog ever been fed entrails and internal organs (Yes, reference: No). How often, if at all, is your dog off-leash? (Often/Mostly/Always, reference: Never/Rarely/Sometimes). Does your dog eat things it finds on the ground while on walks? (Yes, reference: No). How many times, if at all, have you walked your dog (on- or off-leash) in the following areas in the last 6 months? (reference: Minimal (< 3 times a month), 1–6 times/week, daily), and age (years, continuous). The off-leash variable was collapsed from six categories into two because of the lower number of events in each category. Age was treated as non-linear using restricted cubic splines. Knot selection for splines was based off Akaike information criteria.

### Results

A total of 775 surveys were conducted in Canadian dog parks in and around Edmonton, Alberta, Canada. Data consisted of 774 unique fecal samples tested by coproantigen with zinc sulfate centrifugation flotation. Dogs were evenly split by gender, with female dogs representing 48.1% (*n* = 373) of the samples collected. Spayed or neutered dogs made up 89.9% (*n* = 697) of the dogs that participated in the study.

Median age was 4 years, and most dogs (58.6%) were mixed breeds. Based on owners’ responses, 93.4% of dogs had been seen by a veterinarian within the past year; 63.6% of owners indicated that their dog had been dewormed within the past year. Most (60.3%) owners also indicated that given the opportunity, their dogs would chase small prey like rodents, although most ranked their dogs as “unlikely” to be successful in either capturing or eating their prey. Most owners reported that their dogs would scavenge from the ground, with grass and feces the two most common targets at dog parks and while on walks (85.2%).

**Fig. 1:**
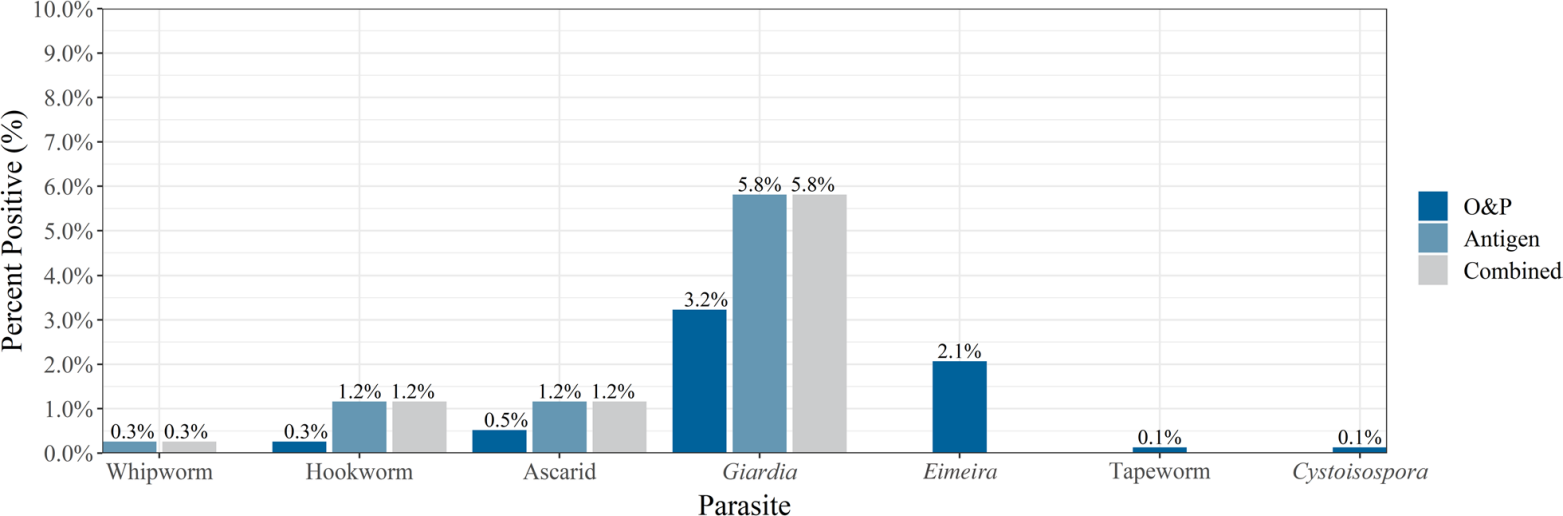
Percent positive for each of the intestinal parasites by method and combined (O&P or antigen)

**Fig. 2:**
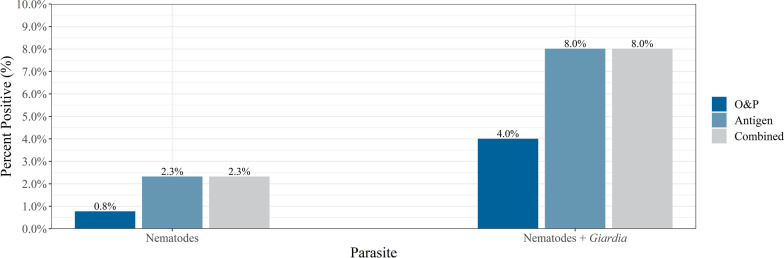
Percent positive for nematodes (hookworm, ascarid, and whipworm) and nematodes plus *Giardia* spp. by method and combined (O&P or antigen)

Coproantigen testing detected more nematode- (ascarid, hookworm, and whipworm) and *Giardia* spp.-positive samples, as well as detecting all of the positive results found by flotation alone (Table 1, Fig. 1). *Giardia* spp. was the most commonly identified intestinal parasite with coproantigen detecting 5.8% (45/774) positive samples compared to flotation, which identified 3.2% (25/774) positive samples (McNemar mid-*P*-value < 0.001). This was followed by ascarids (coproantigen: 1.2%, 9/774, flotation: 0.5%, 4/774, McNemar mid-*P*-value = 0.063) and hookworms (coproantigen: 1.2%, 9/74 flotation: 0.3% 2/774, McNemar mid-*P*-value < 0.016). Whipworm-positive samples were detected by coproantigen (0.3%, 2/774); no whipworm-positive samples were identified using flotation (McNemar mid-*P*-value = 0.500). Tapeworm and *Cystoisospora* had the lowest percentage of positive samples, each with 0.1% detected using flotation (1/774). *Eimeria* spp., a common pseudoparasite that may be confused with *Cystoisospora* spp., was observed in 2.1% (flotation: 2.1%, 16/774) of the samples collected (Table 1). The fecal results showed that 8.0% (*n* = 62) of dogs tested positive for hookworm, ascarid, whipworm, or *Giardia* spp. using any method (Table 1, Fig. 2).

**Table 1:** Percent positive by method and parasite

Parasites	O&P^c^		Antigen		Combined	
	%	*n*/*N*^d^	%	n/*N*^d^	%	n/*N*^d^
Hookworm	0.3	2/774	1.2	9/774	1.2	9/774
Ascarid	0.5	4/774	1.2	9/774	1.2	9/774
Whipworm	–	–	0.3	2/774	0.3	2/774
*Giardia* spp.	3.2	25/774	5.8	45/774	5.8	45/774
*Eimeria* spp.	2.1	16/774	–	–	–	–
Tapeworm	0.1	1/774	–	–	–	–
*Cystoisospora*	0.1	1/774	–	–	–	–
Nematode^a^	0.8	6/774	2.3	18/774	2.3	18/774
Nematode^a^ + *Giardia* spp.	4.0	31/774	8.0	62/774	8.0	62/774
Total^b^	6.3	49/774	8.0	62/774	10.1	78/774

^a^Nematodes include hookworm, ascarid, and whipworm

^b^Total include all parasites and methods combined (including non-infectious parasites)

^c^Ova & parasites: zinc sulfate centrifugal flotation

^d^*n* = total number of positive results; *N* = total number of samples

**Fig. 3:**
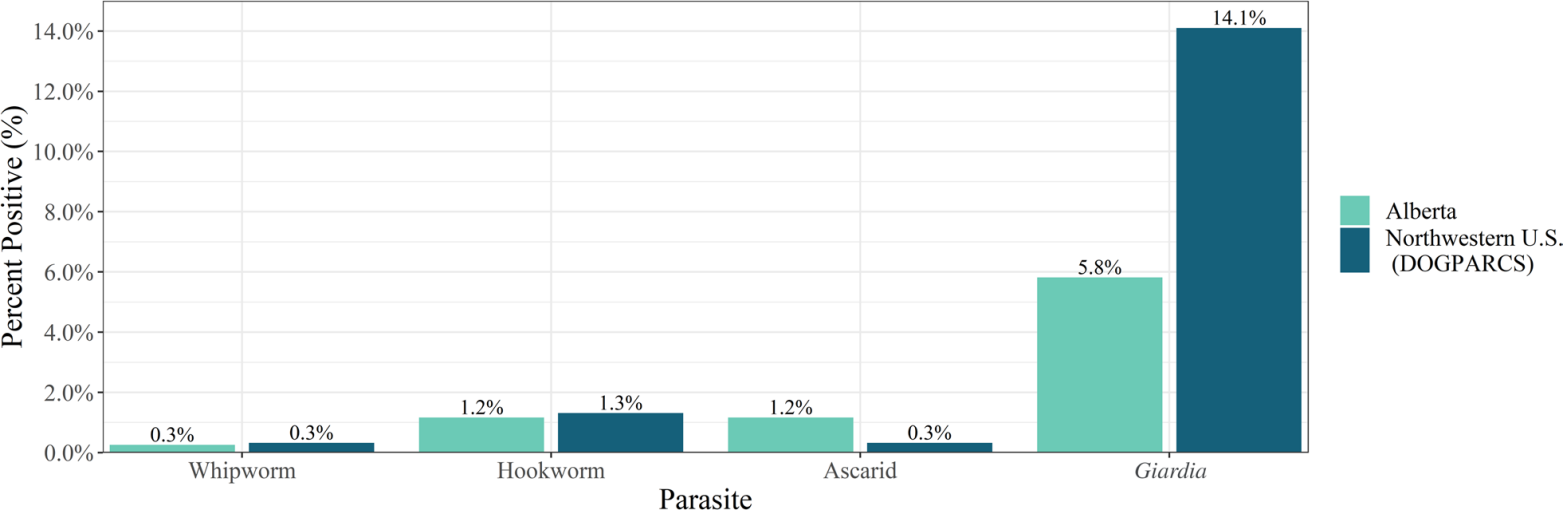
Percent positive for each of the intestinal parasites for combined method (O&P or antigen) between the Northwestern US (Portland, Boise, and Seattle from the DOGPARCs study) compared to samples collected around Alberta, Canada

Combined flotation and coproantigen positivity from the Alberta area were compared with parasite positivity previously reported for three Northwestern cities in the US from the DOGPARCS study (Fig. 3). The combined flotation and coproantigen percent positive was higher in the Northwestern US for *Giardia* spp. whereas the percent of ascarids found in dog parks was higher in Alberta. Similar percent positives in Northwestern US and Alberta were found for hookworms and whipworms (Fig. 3).

Dog owners were surveyed about the frequency of administration of intestinal parasite/heartworm prevention. When questioned about the use of year-round parasite/heartworm prevention, 63.6% stated “yes,” 26.9% stated “no,” and 9.5% stated “unknown.” Owners who stated their dog was on a yearly parasite/heartworm preventative had a combined positivity of 12.4% across both methods and all parasites. Owners who stated their dog was not on a yearly preventative or did not know had a combined positivity of 6.3% and 12.3% across all parasites, respectively.

**Fig. 4:**
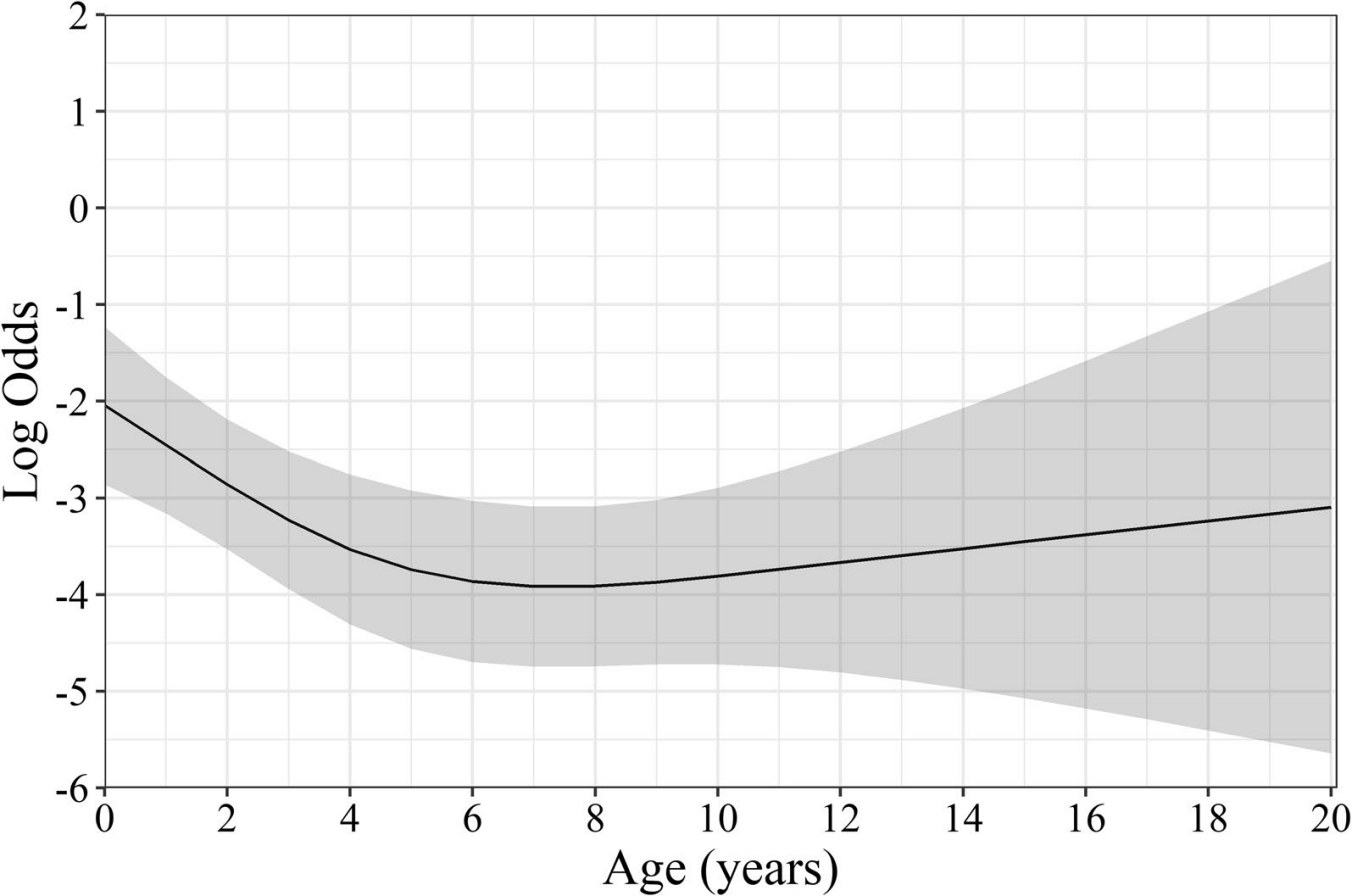
Firth-Penalized regression model showing a non-linear association between any parasite infection and age

A regression model was developed to better understand the factors increasing the odds of having parasite infection. Age was observed to have a non-linear association with parasite infection. Younger dogs had increased odds of having a parasite infection, which steadily decreased until 6 years of age (*P* < 0.001, Fig. 4, Additional file 1: Fig. 1). No effect on the odds of having a parasite infection was observed by level of dog chasing wildlife, eating wildlife entrails, or walking off-leash on in a park (Additional file 1: Fig. 1).

## Discussion

This survey of dogs visiting Edmonton dog parks found that 10.1% (78/774) of all dogs tested positive for at least one parasite, including non-infectious parasites. When comparing flotation and coproantigen testing for the same sample, antigen detected all flotation-positive samples as well as 32 additional positives not detected by flotation alone. When combined with fecal flotation by centrifugation, coproantigen testing optimizes results improving sensitivity over more commonly used flotation methods (CAPC) [1]. Coproantigen testing as part of routine screening may detect sub-clinical and nonpatent infections allowing for appropriate intervention to mitigate environmental contamination, which could lead to ongoing exposure to infection [7].

The overall prevalence of endoparasites was similar to studies in North America [2]. Nematodes (hookworms, whipworms, and ascarids) collectively were present in 2.3% of dogs. Stafford et al. reported values from 2.8% (west) to 17.1% (southeast) depending on the region of the USA where the samples were collected [8]. In a national study conducted in Canada, Villeneuve et al. found higher prevalence of ascarids (8.2%) in Alberta and hookworm (5.6% nationally); however, dogs were surveyed from shelters, and the prevalence is likely higher than in client-owned dogs [9]. In a recent study of client-owned dogs, Smith et al. found an overall prevalence of helminths of 4.1% in Calgary [4]. The variation in these studies suggests that sample source and dog ownership as well as the local ecological conditions play an important role in determining the specific prevalence in a location [9, 10]. The most commonly identified parasite by either coproantigen testing or fecal flotation by centrifugation was *Giardia* spp. in 5.8% of samples. This is comparable (4%) with a large national survey in the US (Little et al.) but lower than that reported in other studies using similar techniques reported by Stafford et al. and Sweet et al. [2, 3, 8]. Reliance on flotation alone, *Giardia* spp. cyst fragility, and demographic factors including samples collected from a variety of sources such as veterinary practices, shelters, and pet stores may have contributed to this wide range. Interestingly the one sample that tested positive for a tapeworm by flotation was the same sample that was determined to be positive for *Echinococcus multilocularis* with a species-specific genetic test that was completed on all the samples [17]. However, the low proportion of intestinal parasite infection may be a result of standard veterinary practices, which typically treat for nematodes not protozoa [1, 4].

Age is known to influence the risk of infections; in particular, young dogs appear to have the highest rates of infection and bear the highest burden of infection [3, 8, 11]. Increased parasite prevalence was also noted in older dogs due to either decreased anthelmintic use or a possible decline in health and immune response [11]. Sample and survey methods may have underrepresented puppies, and as a result the true overall parasite prevalence could be higher. In addition, samples were collected between May and August 2020, suggesting possible seasonal bias. This may be important when considering regional prevalence for hookworms, ascarids, and whipworms, which exhibit differing degrees of seasonal abundance and may be underestimated in some regions during a summer sampling period [13].

Apart from age, this survey did not identify clear risk factors associated with infection. However, other studies have shown that unleashed dogs are more likely to eat/scavenge off the ground and may be more likely to be infected with intestinal parasites [4, 5]. These studies highlighted the risks associated with scavenging and tethering or off-leash activity. Similarly, ownership (stray vs. owned) and geography (urban vs. rural) were risk factors associated with infection [5]. This survey was conducted in an urban setting with client-owned dogs suggesting that owner attention could be a contributing factor to the lower overall parasite prevalence. The ability to further delineate risk factors may be related to the overall lower risk of infection relative to the study conducted by Smith et al. in 2014 [4]. In the Smith study, the rate of infection was sustainably higher for all parasites compared to this sample set, despite the relative proximity of Edmonton and Calgary (~ 300 km). Owners were asked about veterinary history, with 93.4% of owners responding that their dog had received veterinary care within the past year; 63.6% reported their dog had been dewormed in the past year, 26.7% said their dog had not been dewormed in the past year, and 9.7% did not know. However, no clear trends were uncovered despite investigating patterns of infection. Infections appeared more common in the group that was unsure of their dog’s deworming status compared to the group who had self-reported as not deworming their dog in the past year. Self-reporting and social desirability biases may impact individual responses and could suggest more affirmative reporting of veterinary care relative to owners’ actual veterinary visitations [13]. Similarly, confusion over treatment options including management of endo- vs. ectoparasites or terminology (“dewormers” vs. “preventatives”) may have played a role in owners’ responses [8]. Regardless, there is a potential benefit in clear protocols and client education surrounding the care of their pets, in particular, the timing and regularity of some treatments and testing [14].

## Conclusions

Dog parks in Alberta, Canada, showed similar numbers of parasite infections to the dog parks in the northwestern US with *Giardia* spp. infection being lower and ascarid infection being higher. Increasing use of off-leash dog parks may provide important health and socialization benefits for both dog and owners; however, this may come at the cost of increased exposure and transmission of intestinal parasites for dogs and may pose a public health risk for owners. Coproantigen testing detected all flotation-positive samples, as well as 32 additional positives not detected by flotation alone. We encourage routine testing by both flotation and coproantigen and treatment for parasites as well as the collection and disposal of fecal material at parks with proper sanitary stations for owners.

## Supplementary Information

**Additional file 1.**Figure S1. Firth-Penalized regression model showing no evidence of association between odds of parasite infection and owner reported: time off-leash, eating entrails, chasing animals, frequency of park visits, and eating items off ground.

## Data Availability

All data generated or analyzed during this study are included in this published article (and its supplementary information files).
