# Supplementary figures and images for: Canine nematode and Giardia spp. infections in dogs in Edmonton, Alberta, the “CANIDA” study
Source: Parasit Vectors. 2022 Aug 22;15:294. doi: 10.1186/s13071-022-05386-5 (PMC9396815; doi:10.1186/s13071-022-05386-5)

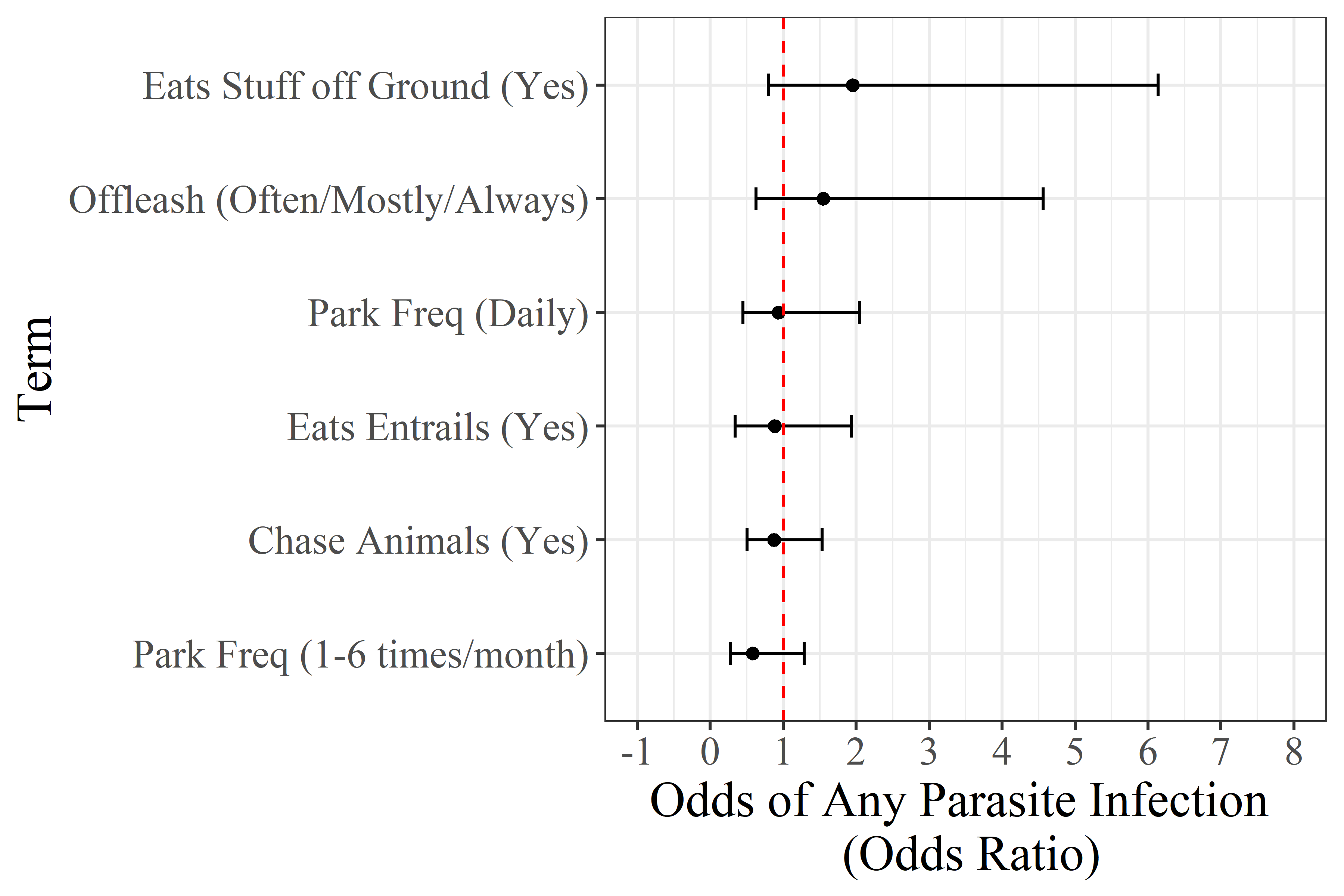

Supplement: Supplementary file 1 — Additional file 1.Figure S1. Firth-Penalized regression model showing no evidence of association between odds of parasite infection and owner reported: time off-leash, eating entrails, chasing animals, frequency of park visits, and eating items off ground. [file 13071_2022_5386_MOESM1_ESM.png]
